# Role of fibrinogen–erythrocyte and erythrocyte–erythrocyte adhesion on cardiovascular pathologies

**DOI:** 10.1080/07853890.2021.1896875

**Published:** 2021-09-28

**Authors:** Filomena A. Carvalho, Ana Filipa Guedes, Luís Sargento, J. Braz Nogueira, Nuno Lousada, Carlos Moreira, Nuno C. Santos

**Affiliations:** a Instituto de Medicina Molecular, Faculdade de Medicina, Universidade de Lisboa, Lisbon, Portugal; b Hospital Pulido Valente, Centro Hospitalar Lisboa Norte, Lisbon, Portugal; c Hospital Santa Maria, Centro Hospitalar Lisboa Norte, Lisbon, Portugal

## Abstract

Cardiovascular pathologies are the major cause of death worldwide. Erythrocyte aggregation is an indicator of cardiovascular risk, which is influenced by high plasma fibrinogen levels. Our main goals were to understand how fibrinogen–erythrocyte binding influences erythrocyte aggregation and how it constitutes a cardiovascular risk factor in essential arterial hypertension (EAH) and chronic heart failure (CHF).

Differences on cell stiffness, protein-cell interaction and cell–cell adhesion forces were evaluated by AFM-based force spectroscopy with cells from 31 EAH patients, 30 CHF patients and 15 healthy blood donors. The main procedures used were previously described by us [1–3]. Results were correlated with patients’ clinical profiles.

From cell–cell adhesion studies, we concluded that, upon increasing fibrinogen concentration (from 0 to 1 mg/mL), there was an increase in the work and force necessary for erythrocyte–erythrocyte detachment on EAH patients and healthy donors. Nevertheless, higher values from both parameters were obtained for EAH patients, when comparing to healthy donors, at each fibrinogen concentration [4].

Fibrinogen-erythrocyte (un)binding forces were higher in EAH and in CHF patients, when compared with the control group, despite a lower binding frequency [5,6]. Ischaemic CHF patients showed increased binding forces compared to non-ischaemic patients. A 12-month clinical follow-up shows that CHF patients with higher fibrinogen–erythrocyte binding forces, probed by AFM at the beginning of the assessment, had a significantly higher probability of being hospitalised due to cardiovascular complications, pointing out the value of AFM for clinical prognosis [5].

Erythrocyte stiffness studies revealed differences between patients and healthy donors, in terms of erythrocyte elasticity (Young’s modulus) and AFM tip penetration depth into the cells [5,6]. Erythrocytes from non-ischaemic CHF patients presented a higher average stiffness than those from the other groups (ischaemic CHF and control). Nevertheless, a significantly higher cell penetration depth at the same applied force was observed for ischaemic CHF patients [5]. In conclusion, fibrinogen promotes erythrocyte adhesion, leading to its aggregation, probably by transient simultaneous binding of the protein to two cells, bridging them. Our results may be relevant for potential future drug interventions to reduce aggregation and enhance microcirculatory flow conditions in cardiovascular patients.
